# Serum progesterone concentration on pregnancy test day might predict ongoing pregnancy after controlled ovarian stimulation and fresh embryo transfer

**DOI:** 10.3389/fendo.2023.1191648

**Published:** 2023-06-26

**Authors:** Marie Duport Percier, Sophie Brouillet, Caroline Mollevi, Martha Duraes, Tal Anahory, Noemie Ranisavljevic

**Affiliations:** ^1^ Department of Reproductive Medicine, Montpellier University Hospital, University of Montpellier, Montpellier, France; ^2^ Department of Reproductive Biology-CECOS, Montpellier University Hospital, University of Montpellier, Montpellier, France; ^3^ Embryo Development Fertility Environment, University of Montpellier, INSERM 1203, Montpellier, France; ^4^ Institute of Epidemiology and Public Health, Montpellier University Hospital, University of Montpellier, INSERM, Montpellier, France

**Keywords:** serum progesterone concentration, luteal phase support, controlled ovarian stimulation, miscarriage, micronized vaginal progesterone, fresh embryo transfer, *in vitro* fertilization (IVF)

## Abstract

Progesterone (P4) is essential for pregnancy. A controlled ovarian stimulation (COS) leads to a iatrogenic luteal defect that indicates a luteal phase support (LPS) at least until pregnancy test day. Some clinicians continue the LPS until week 8 or later, when P4 is mainly secreted by syncytiotrophoblast cells.Measuring serum P4 on pregnancy test day after a fresh embryo transfer could help to identify women who might benefit from prolonged LPS. In women with LPS based on P4 administered by the rectal route, P4 concentration on pregnancy test day was significantly higher in patients with ongoing pregnancy than in patients with abnormal pregnancy.This monocentric retrospective study used data on 99 consecutive cycles of COS, triggered with human chorionic gonadotropin, followed by fresh embryo transfer resulting in a positive pregnancy test (>100 IU/L) (from November 2020 to November 2022). Patients undergoing preimplantation genetic screening or with ectopic pregnancy were excluded. All patients received standard luteal phase support (i.e. micronized vaginal progesterone 600 mg per day for 15 days). The primary endpoint was P4 concentration at day 15 after oocyte retrieval (pregnancy test day) in women with ongoing pregnancy for >12 weeks and in patients with miscarriage before week 12 of pregnancy.The median P4 concentration [range] at pregnancy test day was higher in women with ongoing pregnancy than in women with miscarriage (55.9 ng/mL [11.6; 290.6] versus 18.1 ng/mL [8.3; 140.9], p = 0.002). A P4 concentration ≥16.5 ng/mL at pregnancy test day was associated with higher ongoing pregnancy rate (OR = 12.5, 95% CI 3.61 - 43.33, p <0.001). A P4 concentration ≥16.5 ng/mL at pregnancy test day was significantly associated with higher live birth rate (OR = 11.88, 95% CI 3.30–42.71, p <0.001).After COS and fresh embryo transfer, the risk of miscarriage is higher in women who discontinue luteal support after 15 days, as recommended, but with P4 concentration <16.5 ng/mL. The benefit of individualized prolonged luteal phase support should be evaluated.

## Introduction

During the luteal phase, progesterone, secreted by the corpus luteum, plays a key role in embryo implantation. This hormone is also essential for pregnancy maintenance and for reducing the risk of early miscarriage through immunomodulation and myorelaxation mechanisms (1, 2). Ovarian stimulation for *in vitro* fertilization (IVF) leads to a iatrogenic luteal defect probably secondary to the high sex steroid hormone concentrations (3). Therefore, luteal phase support is required in fresh embryo transfer cycles, but the exact modalities have not been established yet (4). Current data suggest that the ongoing pregnancy rates are not significantly different between women in whom luteal phase support is stopped early (i.e. on pregnancy test day) and in whom luteal phase support is prolonged until week 8 to 12 of gestation (5). Therefore, it is recommended to provide luteal support at least until pregnancy test day (6). However, practice varies among clinicians, and luteal phase support is continued until week 10 or 12 of gestation in most IVF cycles worldwide (7).

In recent years, monitoring serum progesterone (P4) during the luteal phase has become widespread, particularly during artificial cycles for frozen-thawed embryo transfer, where in the absence of corpus luteum, only exogenous progesterone is available (e.g. vaginal micronized progesterone). This simplifies the result interpretation by bypassing the endogenous pulsatile secretion (8). The ideal luteal P4 concentration in artificial cycles with vaginal micronized progesterone is between 10 and 15ng/mL (9). Some authors suggested that in artificial cycles, pregnancy and live birth rates could be improved by individualized luteal phase support (e.g. additional progesterone injections in women with low luteal P4) (10, 11). Conversely, P4 monitoring during the luteal phase of fresh IVF cycles is less frequent. However, some authors have recently shown the predictive value of P4 kinetics during the early luteal phase regarding positive pregnancy test and ongoing pregnancy (12). Yet, measuring serum P4 on pregnancy test day could help to identify women who might benefit from prolonged luteal phase support. Indeed, one prospective study found that in women with luteal phase support based on progesterone administered by the rectal route, P4 concentration at day 15 after oocyte retrieval was significantly higher in patients with ongoing pregnancy than in patients with abnormal pregnancy (median P4 = 135.2 ng/mL, 95% CI 122.6-157.2 ng/mL versus 22.6 ng/mL, 95% CI 15-30.1 ng/mL; p<0.001) (13). These results need to be confirmed, especially in the context of luteal phase support by the vaginal route.

The aim of our study was to evaluate the impact of serum P4 concentration at day 15 after oocyte retrieval (at the time of the first beta human chorionic gonadotropin, hCG, measurement) on the ongoing pregnancy rate in women with positive pregnancy test after ovarian stimulation and fresh embryo transfer with luteal support (15 days of vaginal micronized progesterone).

## Materials and methods

This monocentric retrospective study was carried out at Montpellier University Centre, a tertiary academic hospital, from November 2020 to November 2022.

### Study population

All cycles of ovarian stimulation triggered by hCG and followed by fresh embryo transfer resulting in a positive pregnancy test (beta hCG >100 IU/L) were analysed. Patients were included if data on P4 concentration at day 15 after oocyte retrieval (i.e. pregnancy test day) and ultrasound results at week 12 of pregnancy were available. Patients who received a gonadotropin-releasing hormone (GnRH) agonist trigger (Decapeptyl^®^ 0.2 mg, Ipsen, France), patients with ectopic pregnancy, and patients undergoing IVF for pre-implantation genetic testing were excluded.

Ongoing pregnancy was defined by the observation of foetal cardiac activity on ultrasound after week 12 of gestation. Miscarriage was defined as the loss of an intrauterine pregnancy before week 12 of gestation. Live birth was defined as a live neonate born after 24 weeks of gestation.

### Ovarian stimulation, embryo transfer, and P4 measurement

Ovarian stimulation was performed as previously described (14) using different protocols: long or short agonist protocol (Decapeptyl^®^ 3 mg Ipsen, France; Decapeptyl^®^ 0.1 mg, Ipsen, France; Synarel^®^ 0.4 mg, Pfizer, France), or antagonist protocol (Orgalutran^®^ 0.25 mg, MSD, France) scheduled with luteal oestradiol pre-treatment (Provames^®^ 4mg, Sanofi Aventis, France) or oestrogen-progestin pill (Minidril^®^, Pfizer, France). Ovarian stimulation was performed by daily subcutaneous injection of recombinant follicle-stimulating hormone (Puregon^®^, MSD Organon, OSS The Netherlands; Gonal F^®^, Merck Serono, Geneva, Switzerland; Bemfola^®^, Gedeon Richter, France) or urine-derived follicle-stimulating hormone (Menopur^®^, Ferring Pharmaceuticals, Copenhagen, Denmark; Fertistartkit^®^, Genevrier, France).

Ovulation was triggered with recombinant hCG alfa (Ovitrelle^®^ 250 μg, Serono, Switzerland). Patients received luteal phase support (i.e. micronized progesterone by the vaginal route; Utrogestan^®^ 200mg three times a day, Besins International, SA, France) from the evening of oocyte retrieval until the first pregnancy test at day 15 after oocyte retrieval.

The number of transferred embryos (one or two) was decided by the medical practitioners according to the standard local protocols. Fresh embryo transfer was performed under ultrasound guidance using a Cook^®^ soft catheter at day 3 (D3, cleaved embryo stage) or at D5 (blastocyst stage) or following a double transfer strategy (D2+D4 or D3+D5).

Serum progesterone (P4; ng/mL) and beta hCG were measured at day 15 after oocyte retrieval by the laboratory chosen by the patient. More than 70% of the blood samples were processed using an elecrochemiluminescence immunoassay (Cobas^®^ 8000 801 analyser, Roche Diagnostics, France). Intra- and inter-assay variation coefficients for P4 measurement were ~1.4% and 2%, respectively. Sensitivity was 0.05 ng/ml. Blood sample was achieved 1 to 4 hours after the latest administration of vaginal progesterone.

### Objectives and endpoints

The main objective was to compare P4 concentration on pregnancy test day in patients with ongoing pregnancy and patients with miscarriage before week 12 of gestation.

The primary endpoint was serum progesterone (P4) concentration at day 15 after oocyte retrieval.

The secondary objectives were to define a P4 threshold predictive of ongoing pregnancy and live birth, and to determine whether this was correlated with known factors predictive of miscarriage.

The patient’s age, smoking status, body mass index (BMI), infertility cause (idiopathic, male, female, both), ovarian stimulation protocol, estradiol level (pg/mL) and number of follicles measuring 14mm or more on the day of ovulation trigger or the day before, duration of ovarian stimulation, and number and stage of transferred embryos (cleaved embryos only, blastocysts only, double transfer) were collected. According to the World Health Organization definition, a patient was considered lean if her BMI was <18.5 kg/m2, with normal weight if her BMI was between 18.5 and 25 kg/m2, and with overweight if her BMI was between 25 and 30 kg/m2. Patients with obesity were separated in two groups: moderate obesity (BMI from 30 to 35 kg/m2) and severe obesity (BMI from 35 to 40 kg/m2).

### Statistical analysis

Data were extracted from the BABY SENTRY software and processed using EXCEL.

Qualitative variables were reported as numbers and frequencies, and compared using the Chi-2 test (or Fisher’s exact test if the expected frequencies were <5). Quantitative variables were reported as medians and range (min-max) and compared using Wilcoxon test. All statistical tests were two-sided and the significance level was set at 5% (i.e. p <0.05).

The receiver operating characteristic (ROC) curve was the plot of the true positive rate (= sensitivity) vs false positive rate (= 1 - specificity) for all possible P4 threshold values. The area under the ROC curve (AUC) and its 95% confidence interval (CI) were used as a general measure of the threshold discrimination accuracy between miscarriage and ongoing pregnancy and between live birth and absence of live birth (AUC ≤0.75 = low classification accuracy, 0.75 < AUC < 0.85 = moderate accuracy, and AUC ≥0.85 = high accuracy). The optimal threshold was determined by maximizing the Youden index, defined as J = sensitivity + specificity-1.

A logistic regression model was used to study the association of pregnancy and live birth occurrence with covariates of interest. For each qualitative variable, a reference category was chosen. For continuous variables, the odd ratio (OR) was associated with a one-unit increase. OR were reported with 95% CI. If OR >1, the covariate was favourable; if OR <1, the covariate was deleterious to pregnancy progression. Variables with a (univariate) p value <0.15 were selected for multivariate analysis and a backward covariate selection procedure was performed. All statistical analyses were performed with the Stata 15.0 software.

### Ethical approval

The local research ethics committee reviewed and approved the retrospective collection of clinical and biological data required for this clinical study (Institutional Review Board of Montpellier University Hospital: IRB-MTP_2021_09_202100934).

This research did not receive any specific grant from funding agencies in the public, commercial, or not-for-profit sectors.

The authors declare no conflict of interest.

## Results

**Figure 1:**
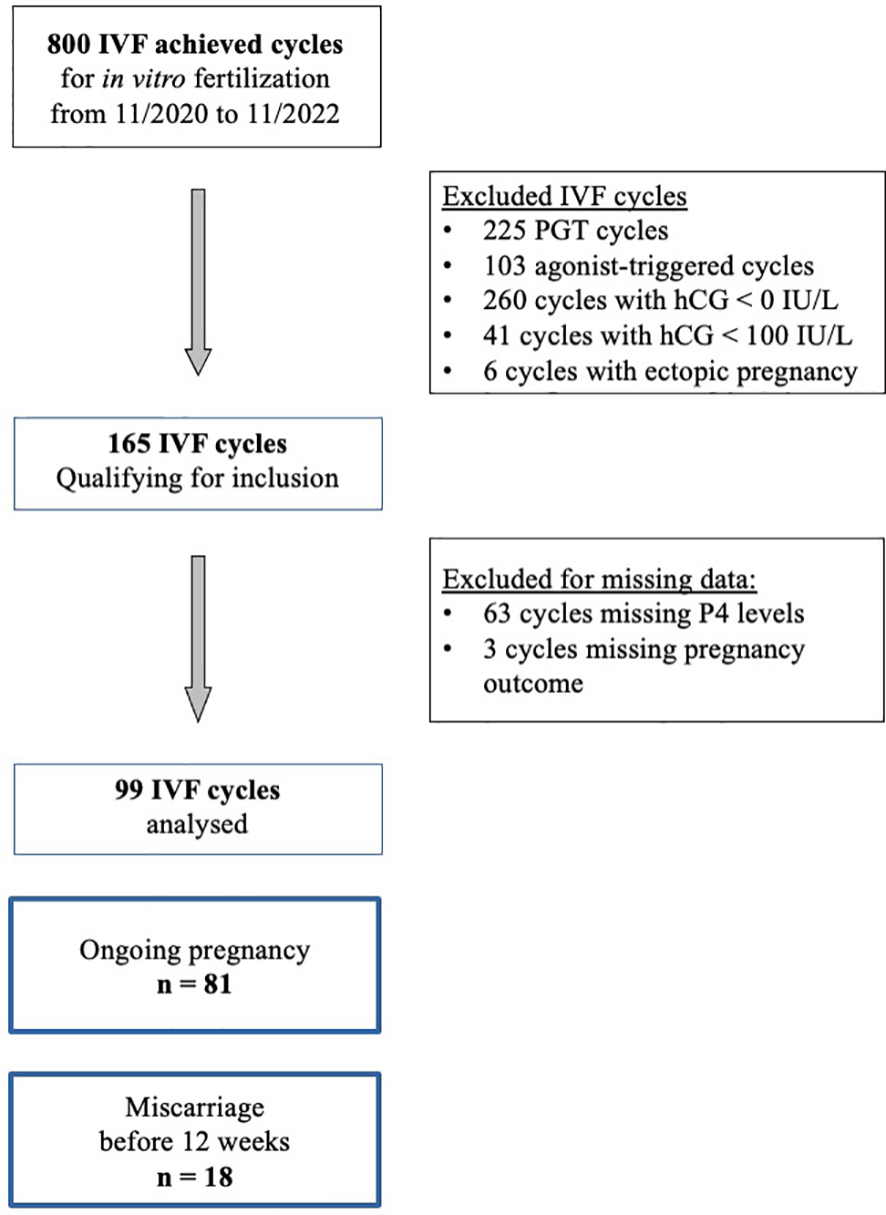
Flow chart.

From November 2020 to November 2022, 800 cycles of ovarian stimulation led to oocyte retrieval for IVF. We excluded 225 cycles performed for preimplantation genetic screening, 103 cycles due to the use of a GnRH agonist trigger, 260 cycles with a negative pregnancy test and 41 cycles with a biochemical pregnancy (beta hCG < 100 IU/L) and 6 cycles due to ectopic pregnancy. In the 165 cycles qualified for inclusion, serum P4 concentration at pregnancy (beta hCG) test at day 15 and ultrasound results were missing for 66 cycles. Finally 99 cycles were retained for the analysis (**Figure 1**).

### Description of the study population

The median age of the included women was 34 years (range: 20 - 43 years). Most patients were less than 35 year-old (58.6%), had normal body weight (61.6%) and were non-smokers (74.7%).

Regarding the cause of infertility, 34% of couples were treated for female infertility, 38% for male infertility, 15% for both, and 13% for unexplained (idiopathic) infertility.

For ovarian stimulation, an antagonist protocol was used in 80% of patients (classic or with mild stimulation). The duration of ovarian stimulation and the ovarian response (estradiol and number of follicles of at least 14mm on trigger day or the day before) were similar in both groups.

One single embryo was transferred in 81% (80/99) of patients (a blastocyst in 51.6% of patients), whereas the double embryo transfer strategy (D3+D5 or D2+D4) was used in the other patients (6%).

Among the 99 included patients with a positive pregnancy test, 18 had a miscarriage before week 12 of pregnancy and the 81 others had an ongoing pregnancy for ≥ 12 weeks. The characteristics of the two groups were comparable (**Table 1**).

**Table 1:** Demographic and clinical characteristics.

		Whole population N=99	Miscarriage N=18	Ongoing pregnancy N=81	p-value
Age (years)					
	*Median (min; max)*	34.00 (20.00; 43.00)	34.50 (25.00; 40.00)	34.00 (20.00; 43.00)	0.377
	< 35	58 (58.59)	9 (50.00)	49 (60.49)	0.626
	[35-40[	24 (24.24)	5 (27.78)	19 (23.46)	
	≥ 40	17 (17.17)	4 (22.22)	13 (16.05)	
BMI (kg/m^2^)					
	*Median (min; max)*	22.80 (17.76; 35.46)	23.34 (17.97; 33.31)	22.77 (17.76; 35.46)	0.959
	Lean	4 (4.65)	1 (6.67)	3 (4.23)	0.815
	Normal weight	53 (61.63)	8 (53.33)	45 (63.38)	
	Overweight	21 (24.42)	5 (33.33)	16 (22.54)	
	Moderate obesity	6 (6.98)	1 (6.67)	5 (7.04)	
	Severe obesity	2 (2.33)	0 (0.00)	2 (2.82)	
	*Missing*	*13*	*3*	*10*	
Smoking status					
	*Median (min; max)*	0.00 (0.00; 35.00)	0.00 (0.00; 35.00)	0.00 (0.00; 20.00)	0.959
	Non-smoker	59 (74.68)	10 (76.92)	49 (74.24)	1.000
	Smoker	20 (25.32)	3 (23.08)	17 (25.76)	
	Non-smoker	59 (74.68)	10 (76.92)	49 (74.24)	0.777
	1 to 9 cigarettes/day	11 (13.92)	1 (7.69)	10 (15.15)	
	>10 cigarettes/day	9 (11.39)	2 (15.38)	7 (10.61)	
	*Missing*	*20*	*5*	*15*	
**Infertility cause**					0.825
	Female	32 (34.04)	7 (41.18)	25 (32.47)	
	Male	36 (38.30)	5 (29.41)	31 (40.26)	
	Idiopathic	12 (12.77)	2 (11.76)	10 (12.99)	
	Mixed	14 (14.89)	3 (17.65)	11 (14.29)	
	*Missing*	*5*	*1*	*4*	
**Ovarian stimulation protocol**					0.190
	Agonist	20 (20.20)	6 (33.33)	14 (17.28)	
	Antagonist	79 (79.80)	12 (66.67)	67 (82.72)	
**Estradiol on trigger day or the day before (pg/mL)**	*Median (min; max)* *Missing*	1652 (564; 3016) *26*	1932 (696; 3014) *5*	1596.5 (564; 3016) *21*	0.453
**Number of follicles ≥14mm on trigger day or the day before**	*Median (min; max)* *Missing*	8 (0; 18) 28	6 (0; 15) 6	8 (1; 18) 22	0.161
**Duration of ovarian stimulation (days)**	*Median (min; max)*	11 (8; 15)	11 (8; 13)	11 (8; 15)	0.415
**Number of transferred embryos**					0.765
	*Median (min; max)*	1.00 (1.00; 2.00)	1.00 (1.00; 2.00)	1.00 (1.00; 2.00)	
**Stage of transferred embryos**					0.790
	Blastocysts	48 (51.61)	8 (47.06)	40 (52.63)	
	Cleaved embryos	45 (48.39)	9 (52.94)	36 (47.37)	
	*Missing*	*6*	*1*	*5*	
**Double transfer**	Yes	6 (6.06)	1 (5.56)	5 (6.17)	1.000

The characteristics of the cycle with missing data (66 cycles) are reported in the **Supplementary Table 1**.

### Serum progesterone concentration and pregnancy outcome

The median [range] P4 concentration at pregnancy test day (day 15 after oocyte retrieval) was higher in the group with ongoing pregnancy ≥ 12 weeks than in the group with miscarriage before week 12 (55.9 ng/mL [11.6; 290.6] versus 18.1 ng/mL [8.3; 140.9], p = 0.002).

**Figure 2:**
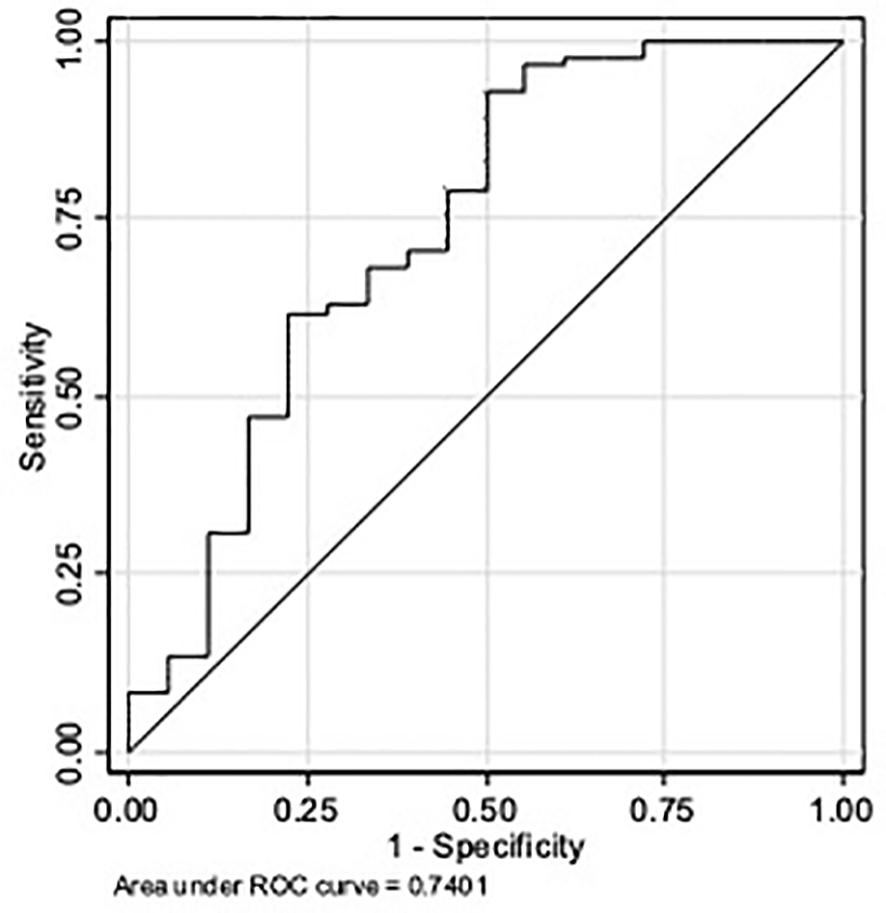
ROC curve for serum P4 concentration as a predictor of pregnancy outcome up to week 12.

The ROC curve analysis of P4 concentration (**Figure 2**) gave an AUC = 0.74 (95% CI 0.64; 0.82), which represents a low accuracy. Based on the Youden index, the optimal P4 cut-off to predict ongoing pregnancy was 16.5 ng/ml (sensitivity of 93%, specificity of 50%, positive predictive value of 89%, negative predictive value of 60%, and overall accuracy of 85.00%).

**Figure 3:**
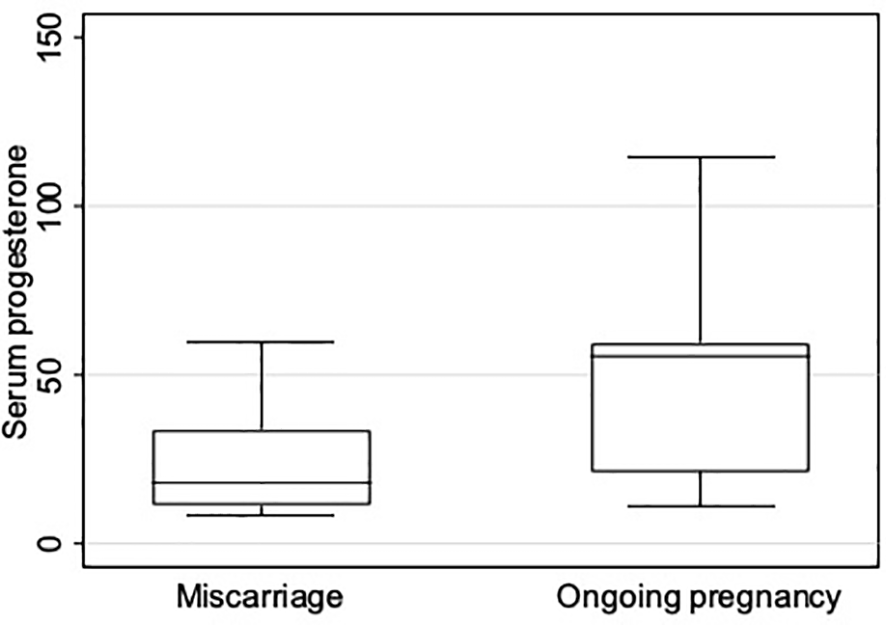
Boxplot showing serum progesterone concentration (ng/mL) distribution on pregnancy test day in patients with miscarriage before week 12 and with ongoing pregnancy after week 12.

The subgroup analysis revealed a significantly different P4 concentration distribution in the two groups (**Table 2**; **Figure 3**). In the group with ongoing pregnancy (n=81), P4 concentration was <16.5ng/ml in 6 women (7.4%) and ≥16.5ng/mL in 75 (92.6%). In the group with miscarriage (n=18), P4 was <16.5ng/ml in 9 (50%) and ≥16.5ng/mL in 9 (50%). (**Table 2**).

**Table 2:** Serum progesterone concentration at day 15 after oocyte retrieval (pregnancy test day).

		Whole population N=99	Miscarriage N=18	Ongoing pregnancy N=81	p-value
**Serum progesterone concentration (ng/ml)**	*Median (min; max)*	41.34 (8.30; 290.56)	18.09 (8.30; 140.90)	55.90 (11.60; 290.6)	**0.002**
	< 11	2 (2.02)	2 (11.11)	0 (0.00)	**<0.001**
	[11-16.5[	13 (13.13)	7 (38.89)	6 (7.41)	**<0.001** **<0.001**
	≥ 16.5	84 (84.85)	9 (50.00)	75 (92.59)	
	< 16.5	15 (15.15)	9 (50.00)	6 (7.41)	
	≥ 16.5	84 (84.85)	9 (50.00)	75 (92.59)	**<0.001**

Significant p value are written in bold.

A P4 concentration ≥16.5 ng/mL at pregnancy test day was significantly associated with higher ongoing pregnancy rate (OR = 12.5, 95% CI 3.61 - 43.33, p <0.001).

A P4 concentration ≥16.5 ng/mL at pregnancy test day was significantly associated with higher live birth rate (OR = 11.88, 95% CI 3.30 – 42.71, p <0.001).

As an indication, the mean P4 concentration among patients with a negative pregnancy test after a fresh embryo transfer during the same period (n=164) was 9.76 ng/mL [0.2; 24.68].

### Additional analyses

The ROC curve analysis of P4 concentration and live birth (**Supplementary Figure 1**) gave an AUC = 0.70 (95% CI 0.59; 0.79), which represents a low accuracy. Based on the Youden index, the optimal P4 cut-off to predict live birth was 16.5 ng/ml (sensitivity of 93%, specificity of 47%, positive predictive value of 87%, negative predictive value of 64%, and overall accuracy of 83.00%).

The logistic regression analysis to study the association of miscarriage or later pregnancy loss with known confounding factors indicated that risk factors of pregnancy loss (patient age, BMI, smoking, cause of infertility, number and stage of transferred embryos) were similar in the two groups (**Supplementary Table 2**). Therefore, the multivariate analysis was not carried out.

Only serum P4 concentration at pregnancy test day was a predictive factor of ongoing pregnancy (OR = 1.02, 95% CI 1.00 -1.04], p=0.028) and tended to be a predictive factor of live birth (OR = 1.01, 95% CI 1.00 -1.03], p=0.055). In other words, there is an estimated 2% and 1% relative increase in odds of ongoing pregnancy and live birth with each 1 ng/mL increment in serum P4 concentration (**Supplementary Table 2**).

## Discussion

This retrospective cohort study indicates that serum progesterone (P4) concentration measured on pregnancy test day (15 days after oocyte retrieval followed by fresh embryo transfer) can predict the pregnancy outcome up to week 12 of gestation (ongoing pregnancy or miscarriage) and the live birth using a threshold of 16.5 ng/mL. This concentration was lower than the threshold proposed by Ioannidis et al. (103 nmol/L, i.e. 32.4 ng/mL), but these thresholds could not be strictly identical because progesterone was administered by the rectal and not vaginal route in this previous study (13). Our threshold is close to the one proposed by Aslih et al. (17 ng/mL) who administered progesterone by the vaginal route, although blood sampling was performed in the mid-luteal phase (day 7 after D2 or D3 embryo transfer) and not on pregnancy test day (15). Despite these methodological differences (route of administration, time of quantification), all three studies suggest that P4 concentration at the end of the luteal phase might predict the pregnancy outcome.

During a natural ovulatory cycle, the corpus luteum, stimulated by endogenous luteinizing hormone (LH), secretes progesterone (16). In early pregnancy, hCG takes over and promotes P4 secretion by the corpus luteum. Then, from the beginning of week 8 of gestation, P4 is mainly secreted by syncytiotrophoblast cells (17). In stimulated IVF cycles, the development of a large number of follicles leads to the formation of many corpora lutea after ovulation, and consequently to elevated P4 secretion that inhibits LH secretion through a negative feedback (18). Thus, after hCG trigger, P4 concentration first increases until day 4 and then drops to zero at day 8-10 after oocyte retrieval (19). The aim of the luteal phase support is to alleviate this “luteal gap” between the stimulatory effects of exogenous hCG (used to trigger ovulation) and of endogenous hCG from the conceptus. We can hypothesize that the patients in our cohort with P4 concentration <16.5 ng/mL on pregnancy test day presented a severe iatrogenic luteal defect with major luteolysis and their corpora lutea could not properly resume progesterone secretion in response to hCG from the early pregnancy. Serum P4 levels measured during a stimulated IVF cycle reflect both the exogenous and endogenous P4 contribution. According to studies on artificial cycles without corpus luteum, absorption of progesterone delivered by the vaginal route usually results in a serum P4 concentration of ~10-15 ng/mL (9). Therefore, P4 concentrations <16.5 ng/mL might reflect the absorption of exogenous vaginal progesterone in the absence of functional corpora lutea, or at least in the presence of insufficiently functional corpora lutea. Identifying patients with insufficiently functional corpora lutea as early as during the mid-luteal phase would be of interest as suggested in a recent study: the drop of P4 from day 3 after oocyte retrieval to day 5 after oocyte retrieval appears to be predictive of ongoing pregnancy, mainly due to a lower initial pregnancy rate rather than an increased pregnancy loss rate (12). In this study, luteal phase support was not interrupted on pregnancy test day and authors did not analyze the data separately for patients with a positive pregnancy test only. It would be interesting to assess if the patients with a low P4 on the day of a positive pregnancy test displayed a drop in P4 in the early to mid-luteal phase. This would comfort the hypothesis of insufficiently functional corpora lutea.

In our study, two groups were defined according to pregnancy progression up to the week 12 of gestation (ongoing pregnancy or miscarriage). We wanted to determine whether miscarriage before week 12 could be explained by low P4 concentration due to severe luteolysis or non-functional corpora lutea in early pregnancy, after discontinuation of luteal phase support. However, we could not exclude an embryonic cause of miscarriage, although most women in our study were rather young. Indeed, pre-implantation genetic testing for aneuploidy (PGT-A) is not allowed in France and some patients in our cohort might have had an early miscarriage related to undiagnosed aneuploidy. However, as PGT-A most often requires a freeze-all strategy while waiting for the genetic test results (20), it cannot be used in studies in which IVF is followed by fresh embryo transfer. It is therefore difficult to design a study that would investigate the impact of low P4 levels on the day of the pregnancy test following a fresh euploid embryo transfer. While there appears to be an association between P4 levels and pregnancy outcomes, causality has not yet been demonstrated. Indeed, it is impossible to distinguish between a low P4 level secondary to luteal insufficiency causing a miscarriage and a pregnancy with unfavourable evolution causing insufficient stimulation of the corpora lutea and resulting in a low P4 level.

If the finding about the usefulness of serum P4 measurement on pregnancy test day to assess the efficiency of corpus luteum rescue by the ensuing pregnancy after ovarian stimulation is confirmed, it could be used to guide the decision of prolonging luteal phase support in a subpopulation of patients. A recent literature review suggests that luteal phase support duration might be individualized (21). In a retrospective cohort study, Segal et al. reported that the duration of luteal phase support could be adjusted in function of P4 and oestradiol concentrations on pregnancy test day (22). Specifically, progesterone supplementation was stopped on pregnancy test day if oestradiol and P4 concentrations were ≥272.4 pg/ml and ≥ 34.5 ng/ml, respectively. Otherwise, treatment was continued until week 7 of gestation. They found similar live birth and miscarriage rates in both groups (with and without prolonged progesterone supplementation), suggesting that in women with strong endogenous luteal activity, exogenous luteal support could be stopped on pregnancy test day, without affecting pregnancy outcome.

There is no strong evidence in the literature to suggest a deleterious effect of longer progesterone supplementation during early pregnancy. Some studies suggest that excessive serum P4 in the late follicular phase (23) or even in the mid-luteal phase of fresh embryo transfer cycles (24) and artificial cycles (25) may be associated with lower pregnancy rates. Moreover, the question arises as to whether maintaining the luteal phase support when corpora lutea secrete enough P4 could be deleterious. In any case, stopping a useless luteal phase support would reduce the treatment burden for patients.

Although a multiple regression analysis was performed, the main limitations of our study are its small size, its retrospective nature, and single-centre design. However, despite a small sample size, we observed a statistically significant difference in this pilot study. There could be some confounding factors that may alter the absorption of progesterone delivered by the vaginal route, such as sexual intercourse (26) and obesity (27). Data were missing in a large number of cycles (66 cycles) which may introduce a selection bias, although patient and cycle characteristics appear to be similar (**Supplementary Table 1**). Moreover, patient compliance with progesterone administration was not recorded, nor was the hour of P4 measurement nor the precise delay between last progesterone administration and blood collection. Lastly, patients could perform the pregnancy test and serum P4 measurement in the laboratory of their choice. Variability between P4 assay kits has been described; nevertheless, these variations are larger for extreme P4 values and smaller for average values, which was the case for our cohort (28). Therefore, the threshold of P4 proposed in our study should be interpreted with caution. Moreover, P4 thresholds depend on the type, route and dose of progesterone used for luteal phase support and might need to be adjusted according to the local practice.

Despite these limitations, this study is the first to report that after fresh embryo transfer and 2-week vaginal luteal support, P4 concentration on pregnancy test day can predict pregnancy outcome. This finding raises questions about the possible benefit of individualized prolonged luteal phase support. Based on our results, it would be interesting to assess the benefit of a personalized luteal phase support protocol for patients with positive pregnancy test after fresh embryo transfer in function of their serum P4 value on pregnancy test day:

- very low P4 (i.e. <10 ng/mL): obvious luteal insufficiency and suspected poor vaginal absorption;
- low P4 (i.e. 10 ng/mL < P4 < 16.5 ng/mL): possible luteal insufficiency and/or poor vaginal absorption;
- high P4 (I.e. P4 ≥16.5ng/mL): based on our study, luteal support seems to be unnecessary and could be stopped on pregnancy test day since the corpora lutea appear to have taken over the secretion of progesterone.

Prospective studies are needed to determine the exact modalities of P4 monitoring and the optimal luteal phase support and its individualization. Such studies would help to better understand the relationship between P4 levels on pregnancy test day after a fresh embryo transfer and pregnancy outcome, as well as to identify a possible causality between low P4 level and miscarriage.

## Data availability statement

The raw data supporting the conclusions of this article will be made available by the authors, without undue reservation.

## Ethics statement

The studies involving human participants were reviewed and approved by Institutional Review Board (I.R.B.) MONTPELLIER UNIVERSITY HOSPITAL. Written informed consent for participation was not required for this study in accordance with the national legislation and the institutional requirements.

## Author contributions

MDP, CM, TA and NR participated in study design. All authors participated in study execution. MDP, CM and NR participated in analysis. MDP, CM, SB and NR participated in manuscript drafting. All authors participated in critical discussion. All authors contributed to the article and approved the submitted version.

## References

[1] Di Renzo GC Giardina I Clerici G Brillo E Gerli S. Progesterone in normal and pathological pregnancy. Horm Mol Biol Clin Investig (2016) 27:35–48. doi: 10.1515/hmbci-2016-0038 27662646

[2] Csapo AI Pulkkinen M. Indispensability of the human corpus luteum in the maintenance of early pregnancy *Luteectomy evidence* . Obstet Gynecol Surv (1978) 33:69–81. doi: 10.1097/00006254-197802000-00001 341008

[3] Beckers NGM Macklon NS Eijkemans MJ Ludwig M Felberbaum RE Diedrich K. Nonsupplemented luteal phase characteristics after the administration of recombinant human chorionic gonadotropin, recombinant luteinizing hormone, or gonadotropin-releasing hormone (GnRH) agonist to induce final oocyte maturation in *in vitro* fertilization patients after ovarian stimulation with recombinant follicle-stimulating hormone and GnRH antagonist cotreatment. J Clin Endocrinol Metab (2003) 88:4186–92. doi: 10.1210/jc.2002-021953 12970285

[4] van der Linden M Buckingham K Farquhar C Kremer JAM Metwally M. Luteal phase support for assisted reproduction cycles. Cochrane Database Syst Rev (2015) 2015(7):CD009154. doi: 10.1002/14651858.CD009154.pub3 26148507 PMC6461197

[5] Liu X-R Mu H-Q Shi Q Xiao X-Q Qi H-B. The optimal duration of progesterone supplementation in pregnant women after IVF/ICSI: a meta-analysis. Reprod Biol Endocrinol RBE (2012) 10:107. doi: 10.1186/1477-7827-10-107 PMC3551800 23237065

[6] Ovarian Stimulation TEGGO Bosch E Broer S Griesinger G Grynberg M Humaidan P. ESHRE guideline: ovarian stimulation for IVF/ICSI†. Hum Reprod Open (2020) 2020:hoaa009. doi: 10.1093/hropen/hoaa009 32395637 PMC7203749

[7] Vaisbuch E Leong M Shoham Z. Progesterone support in IVF: is evidence-based medicine translated to clinical practice? a worldwide web-based survey. Reprod BioMed Online (2012) 25:139–45. doi: 10.1016/j.rbmo.2012.04.005 22683150

[8] Ranisavljevic N Huberlant S Montagut M Alonzo P-M Darné B Languille S. Low luteal serum progesterone levels are associated with lower ongoing pregnancy and live birth rates in ART: systematic review and meta-analyses. Front Endocrinol (2022) 13:892753. doi: 10.3389/fendo.2022.892753 PMC9229589 35757393

[9] Melo P Chung Y Pickering O Price MJ Fishel S Khairy M. Serum luteal phase progesterone in women undergoing frozen embryo transfer in assisted conception: a systematic review and meta-analysis. Fertil Steril (2021) 116:1534–56. doi: 10.1016/j.fertnstert.2021.07.002 34384594

[10] Labarta E Mariani G Rodríguez-Varela C Bosch E. Individualized luteal phase support normalizes live birth rate in women with low progesterone levels on the day of embryo transfer in artificial endometrial preparation cycles. Fertil Steril (2022) 117:96–103. doi: 10.1016/j.fertnstert.2021.08.040 34548167

[11] Álvarez M Gaggiotti-Marre S Martínez F Coll L García S González-Foruria I. Individualised luteal phase support in artificially prepared frozen embryo transfer cycles based on serum progesterone levels: a prospective cohort study. Hum Reprod Oxf Engl (2021) 36(6):1552–60. doi: 10.1093/humrep/deab031 33686413

[12] Uyanik E Mumusoglu S Polat M Yarali Ozbek I Esteves SC Humaidan P. A drop in serum progesterone from oocyte pick-up +3 days to +5 days in fresh blastocyst transfer, using hCG-trigger and standard luteal support, is associated with lower ongoing pregnancy rates. Hum Reprod Oxf Engl (2023) 38:225–36. doi: 10.1093/humrep/deac255 36478179

[13] Ioannidis G Sacks G Reddy N Seyani L Margara R Lavery S. Day 14 maternal serum progesterone levels predict pregnancy outcome in IVF/ICSI treatment cycles: a prospective study. Hum Reprod Oxf Engl (2005) 20:741–6. doi: 10.1093/humrep/deh644 15591085

[14] Ranisavljevic N Hess M Castelli C Willems M Ferrieres-Hoa A Girardet A. Are ovarian response and pregnancy rates similar in selected FMR1 premutated and mutated patients undergoing preimplantation genetic testing? J Assist Reprod Genet (2020) 37:1675–83. doi: 10.1007/s10815-020-01809-3 PMC7376994 32483686

[15] Aslih N Ellenbogen A Shavit T Michaeli M Yakobi D Shalom-Paz E. Can we alter pregnancy outcome by adjusting progesterone treatment at mid-luteal phase: a randomized controlled trial. Gynecol Endocrinol Off J Int Soc Gynecol Endocrinol (2017) 33:602–6. doi: 10.1080/09513590.2017.1298742 28277886

[16] Filicori M Butler JP Crowley WF. Neuroendocrine regulation of the corpus luteum in the human. evidence for pulsatile progesterone secretion. J Clin Invest (1984) 73:1638–47. doi: 10.1172/JCI111370 PMC437074 6427277

[17] Kawachiya S Bodri D Hirosawa T Yao Serna J Kuwahara A Irahara M. Endogenous progesterone levels could predict reproductive outcome in frozen embryo replacement cycles supplemented with synthetic progestogens: a retrospective cohort study. Reprod Med Biol (2019) 18:91–6. doi: 10.1002/rmb2.12254 PMC6332737 30655726

[18] Fatemi HM. The luteal phase after 3 decades of IVF: what do we know? Reprod BioMed Online (2009) 19 Suppl 4:4331. doi: 10.1016/S1472-6483(10)61065-6 20034417

[19] Connell MT Szatkowski JM Terry N DeCherney AH Propst AM Hill MJ. Timing luteal support in assisted reproductive technology: a systematic review. Fertil Steril (2015) 103:939–946.e3. doi: 10.1016/j.fertnstert.2014.12.125 25638420 PMC4385437

[20] Sciorio R Tramontano L Catt J. Preimplantation genetic diagnosis (PGD) and genetic testing for aneuploidy (PGT-a): status and future challenges. Gynecol Endocrinol Off J Int Soc Gynecol Endocrinol (2020) 36:6–11. doi: 10.1080/09513590.2019.1641194 31317806

[21] Mizrachi Y Raziel A Weissman A. When can we safely stop luteal phase support in fresh IVF cycles? a literature review. Front Reprod Health (2020) 2:610532. doi: 10.3389/frph.2020.610532 36304703 PMC9580666

[22] Segal L Breyzman T Kol S. Luteal phase support post IVF: individualized early stop. Reprod BioMed Online (2015) 31:633–7. doi: 10.1016/j.rbmo.2015.07.011 26371712

[23] Venetis CA Kolibianakis EM Bosdou JK Tarlatzis BC. Progesterone elevation and probability of pregnancy after IVF: a systematic review and meta-analysis of over 60 000 cycles. Hum Reprod Update (2013) 19:433–57. doi: 10.1093/humupd/dmt014 23827986

[24] Thomsen LH Kesmodel US Erb K Bungum L Pedersen D Hauge B. The impact of luteal serum progesterone levels on live birth rates-a prospective study of 602 IVF/ICSI cycles. Hum Reprod Oxf Engl (2018) 33:1506–16. doi: 10.1093/humrep/dey226 29955789

[25] Yovich JL Conceicao JL Stanger JD Hinchliffe PM Keane KN. Mid-luteal serum progesterone concentrations govern implantation rates for cryopreserved embryo transfers conducted under hormone replacement. Reprod BioMed Online (2015) 31:180–91. doi: 10.1016/j.rbmo.2015.05.005 26099447

[26] Merriam KS Leake KA Elliot M Matthews ML Usadi RS Hurst BS. Sexual absorption of vaginal progesterone: a randomized control trial. Int J Endocrinol (2015) 2015:685281. doi: 10.1155/2015/685281 25713585 PMC4332976

[27] Bellver J Rodríguez-Varela C Brandão P Labarta E. Serum progesterone concentrations are reduced in obese women on the day of embryo transfer. Reprod BioMed Online (2022) 45:679–87. doi: 10.1016/j.rbmo.2022.05.022 35843779

[28] Boudou P Taieb J Mathian B Badonnel Y Lacroix I Mathieu E. Comparison of progesterone concentration determination by 12 non-isotopic immunoassays and gas chromatography/mass spectrometry in 99 human serum samples. J Steroid Biochem Mol Biol (2001) 78:97–104. doi: 10.1016/s0960-0760(01)00078-4 11530290

