# Supplementary figures and images for: Serum progesterone concentration on pregnancy test day might predict ongoing pregnancy after controlled ovarian stimulation and fresh embryo transfer
Source: Front Endocrinol (Lausanne). 2023 Jun 26;14:1191648. doi: 10.3389/fendo.2023.1191648 (PMC10338216; doi:10.3389/fendo.2023.1191648)

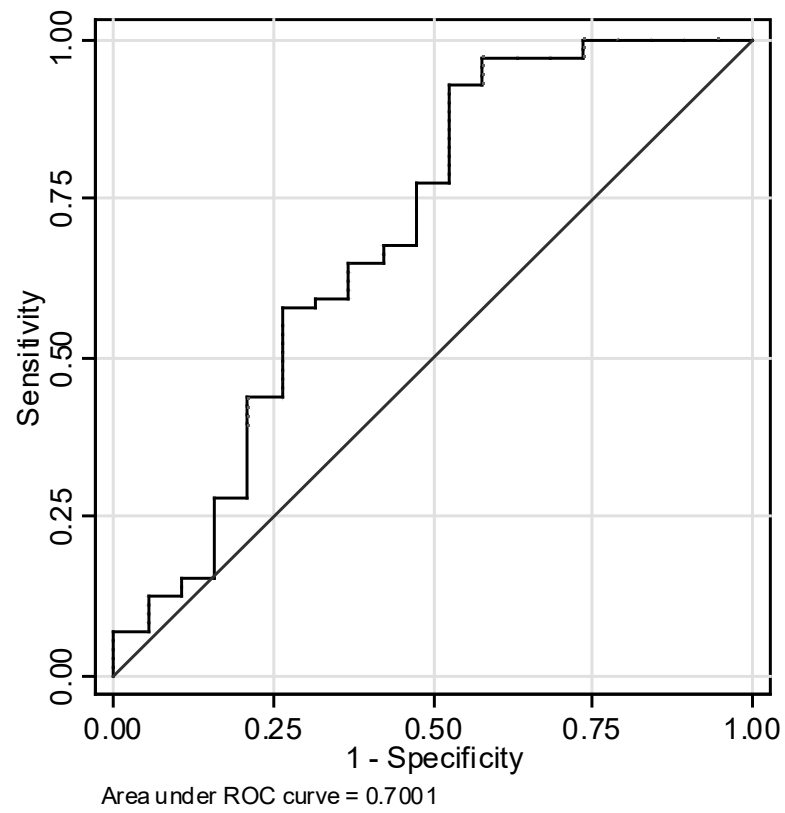

Supplementary Figure 1  
ROC curve for serum P4 concentration as a predictor of live birth.

Supplement: Supplementary file 1 [file Image_1.pdf]
