# Supplementary material for: Serum progesterone concentration on pregnancy test day might predict ongoing pregnancy after controlled ovarian stimulation and fresh embryo transfer
Source: Front Endocrinol (Lausanne). 2023 Jun 26;14:1191648. doi: 10.3389/fendo.2023.1191648 (PMC10338216; doi:10.3389/fendo.2023.1191648)
Supplement: Supplementary file 2 [file Table_1.docx]

*Supplementary Table 1. Logistic model*

|  |  | **Miscarriage vs Ongoing pregnancy**  **Univariate (N=99)** | | | **No live birth vs Live birth**  **Univariate (N=90)** | | |
| --- | --- | --- | --- | --- | --- | --- | --- |
|  |  | **OR** | **95% CI** | **p-value** | **OR** | **95% CI** | **p-value** |
| **Age (years)** |  |  | | 0.451 |  | | 0.168 |
|  | *(increase of 1 unit)* | 0.96 [ 0.85 - 1.07 ] | |  | 0.92 [ 0.82 - 1.04 ] | |  |
|  | < 35 | 1 | | 0.704 | 1 | | 0.374 |
|  | [35-40[ | 0.70 [ 0.21 - 2.35 ] | |  | 0.64 [ 0.19 - 2.20 ] | |  |
|  | ≥ 40 | 0.60 [ 0.16 - 2.25 ] | |  | 0.40 [ 0.11 - 1.45 ] | |  |
| **BMI (kg/m^2^)** |  |  | | 0.915 |  | | 0.998 |
|  | *(increase of 1 unit)* | 0.99 [ 0.88 - 1.13 ] | |  | 1.00 [ 0.88 - 1.14 ] | |  |
| **Smoking status** |  |  | | 0.181 |  | | 0.244 |
|  | *(increase of 1 unit)* | 0.94 [ 0.87 - 1.02 ] | |  | 0.95 [ 0.88 - 1.03 ] | |  |
|  | Non-smoker | 1 | | 0.838 | 1 | | 0.737 |
|  | Smoker | 1.16 [ 0.28 - 4.70 ] | |  | 1.27 [ 0.31 - 5.22 ] | |  |
|  | Non-smoker | 1 | | 0.700 | 1 | | 0.722 |
|  | 1 to 9 cigarettes/day | 2.04 [ 0.23 - 17.79 ] | |  | 2.14 [ 0.24 - 18.92 ] | |  |
|  | >10 cigarettes/day | 0.71 [ 0.13 - 3.96 ] | |  | 0.83 [ 0.15 - 4.64 ] | |  |
| **Infertility cause** |  |  | | 0.833 |  | | 0.705 |
|  | Female | 1 | |  | 1 | |  |
|  | Male | 1.74 [ 0.49 - 6.14 ] | |  | 2.03 [ 0.56 - 7.31 ] | |  |
|  | Idiopathic | 1.40 [ 0.25 - 7.93 ] | |  | 1.05 [ 0.22 - 5.02 ] | |  |
|  | Mixed | 1.03 [ 0.22 - 4.73 ] | |  | 1.17 [ 0.25 - 5.50 ] | |  |
| **Ovarian stimulation protocol** |  |  | | **0.144** |  | | **0.129** |
|  | Agonist | 1 | |  | 1 | |  |
|  | Antagonist | 2.39 [ 0.77 - 7.46 ] | |  | 2.52 [ 0.79 - 8.04 ] | |  |
| **Number of transferred embryos** |  |  | | 0.760 |  | | 0.898 |
|  | *(increase of 1 unit)* | 1.23 [ 0.32 - 4.77 ] | |  | 0.92 [ 0.26 - 3.21 ] | |  |
| **Stage of transferred embryos** |  |  | | 0.678 |  | | 0.786 |
|  | Blastocysts | 1 | |  | 1 | |  |
|  | Cleaved embryos | 0.80 [ 0.28 - 2.29 ] | |  | 0.86 [ 0.30 - 2.51 ] | |  |
| **Serum progesterone concentration (ng/ml)** |  |  | | **0.028** |  | | **0.055** |
|  | (increase of 1 unit) | 1.02 [ 1.00 - 1.04 ] | |  | 1.01 [ 1.00 - 1.03 ] | |  |
|  | < 16.5 | 1 | | **<0.001** | 1 | | **<0.001** |
|  | ≥ 16.5 | 12.50 [ 3.61 - 43.33 ] | |  | 11.88 [ 3.30 - 42.71 ] | |  |
