# Supplementary material for: Serum progesterone concentration on pregnancy test day might predict ongoing pregnancy after controlled ovarian stimulation and fresh embryo transfer
Source: Front Endocrinol (Lausanne). 2023 Jun 26;14:1191648. doi: 10.3389/fendo.2023.1191648 (PMC10338216; doi:10.3389/fendo.2023.1191648)
Supplement: Supplementary file 3 [file Table_2.docx]

Supplementary Table 2. Demographic and clinical characteristics

|  | | **Whole population**  **N=99** | **Missing data**  **N=66** |
| --- | --- | --- | --- |
| **Age (years)** |  |  |  |
|  | *Median (min; max)* | 34.00 (20.00 ; 43.00) | 34.50 (20.00 ; 40.00) |
|  | < 35 | 58 (58.59) | 33 (50.00) |
|  | [35-40[ | 24 (24.24) | 27 (40.91) |
|  | ≥ 40 | 17 (17.17) | 6 (9.09) |
| **BMI (kg/m^2^)** |  |  |  |
|  | *Median (min; max)* | 22.80 (17.76 ; 35.46) | 22.58 (16.81 ; 38.87) |
|  | Lean | 4 (4.65) | 7 (11.11) |
|  | Normal weight | 53 (61.63) | 36 (57.14) |
|  | Overweight | 21 (24.42) | 11 (17.46) |
|  | Moderate obesity | 6 (6.98) | 7 (11.11) |
|  | Severe obesity | 2 (2.33) | 2 (3.17) |
|  | *Missing* | *13* | *3* |
| **Smoking status** |  |  |  |
|  | *Median (min; max)* | 0.00 (0.00 ; 35.00) | 0.00 (0.00 ; 20.00) |
|  | Non-smoker | 59 (74.68) | 42 (76.36) |
|  | Smoker | 20 (25.32) | 13 (23.64) |
|  | Non-smoker | 59 (74.68) | 42 (76.36) |
|  | 1 to 9 cigarettes/day | 11 (13.92) | 5 (9.09) |
|  | >10 cigarettes/day | 9 (11.39) | 8 (14.55) |
|  | *Missing* | *20* | *11* |
| **Infertility cause** |  |  |  |
|  | Female | 32 (34.04) | 27 (44.26) |
|  | Male | 36 (38.30) | 19 (31.15) |
|  | Idiopathic | 12 (12.77) | 7 (11.48) |
|  | Mixed | 14 (14.89) | 8 (13.11) |
|  | *Missing* | *5* | *5* |
| **Ovarian stimulation protocol** |  |  |  |
|  | Agonist | 20 (20.20) | 18 (27.27) |
|  | Antagonist | 79 (79.80) | 48 (72.73) |
| **Estradiol on trigger day or the day before (pg/mL)** | *Median (min; max)*  *Missing* | 1652 (564 ; 3016)  *26* | 2106.5 (993.8 ; 3985)  *18* |
| **Number of follicles ≥14mm on trigger day or the day before** | *Median (min; max)*  *Missing* | 8 (0 ; 18)  *28* | 9 (2 ; 16)  *19* |
| **Duration of ovarian stimulation (days)** | *Median (min; max)* | 11 (8 ; 15) | 11 (8 ; 16) |
| **Number of transferred embryos** |  |  |  |
|  | *Median (min; max)* | 1.00 (1.00 ; 2.00) | 1.00 (1.00 ; 2.00) |
| **Stage of transferred embryos** |  |  |  |
|  | Blastocysts | 48 (51.61) | 36 (58.06) |
|  | Cleaved embryos | 45 (48.39) | 26 (41.94) |
|  | *Missing* | *6* | *4* |
| **Double transfer** | Yes | 6 (6.06) | 4 (6.06) |
